# In This Issue

**DOI:** 10.1002/cfg.71

**Published:** 2001-02


**Comparative and Functional Genomics**
*Comp Funct Genom* 2001; **2**: 1–2.

### In this Issue

### Comparison of NCBI and WU-BLAST for single-pass sequence homology searches

In their short communication, Woodwark *et al.* describe a comparison of the performance of NCBI and Washington University BLASTn tools for detecting homologies for single-pass sequences. This type of input data typically includes single base errors, which cause gaps in homologies. The authors describe their observations of a difference in the way that the these two programmes deal with this problem, which leads to the conclusion that, in this case at least, one outperforms the other.

In this issue we feature the second Agricultural Microbes Genome meeting (AMG2: **http://www.intl-pag.org/amg**), sponsored by the USDA. Until fairly recently, the emphasis in bacterial sequencing has been firmly placed upon human pathogens. However, pathogens and symbionts of farm animals and plants play an important role in agriculture and are also worthy of intense study. The aim of this meeting, then, is to bring this community together and to focus attention on their efforts.

### Conference editorial

Peter Johnson, Noel Keen, Joan Lunney and Michael Sadowsky provide us with an introduction to the Second Agricultural Microbes Genome Meeting, held in San Diego in January 2001. They describe the reasoning behind the meeting and give a background to each of the presentations made in the three main sessions.

### Conference papers

### Microbial comparative genomics

Comparative genomics is a particularly powerful tool for the analysis of bacterial conserved hypothetical proteins, since over 30 complete bacterial genomes are publicly available. In his review, Michael Galperin discusses the strengths and limitations of applying such computational approaches to the assignment of function of these proteins.

### Analysis of the *Cryptosporidium parvum* genome

Mitchell Abrahamsen reviews the projects that have been undertaken, and those that are currently under way, in the study of the *Cryptosporidium parvum* genome, in particular the sequencing project. The project will provide valuable data for treating and preventing the diseases caused by this important pathogen, but also for comparative analyses, e.g. with the genome of *Plasmodium falciparum*, which is nearing completion.

### Functional analysis of *Bacillus subtilis*

*Bacillus subtilis* is a well-established model for Gram-positive bacteria and has been extensively studied. Colin Harwood and colleagues describe the wide range of projects that are being aimed at assigning function to the genes revealed by the complete sequencing of the genome in 1997, one-third of which currently have no known function.

### Bioinformatics

Peter Karp illustrates for us a problem that is the bane of the lives of those who write software to search sequence databases. While the structure that has been laid down for Genbank entries is a good one, and the concept of having entries which are consistent is laudable, it appears that it is not an easy thing to police. Already, there are several entries, including some from whole genome sequencing consortia that do not conform to the standard, rendering search tools unable to locate certain fields of information.

### Microarray data analysis—pattern recognition and prediction

Several groups have already been applying microarray technology to the classification of samples, for

Copyright © 2001 John Wiley & Sons, Ltd.

example, into different types of cancers. One factor that can limit the ability to accurately classify samples is the small sample size available in each case. Edward Dougherty reviews the considerations for selection of classifiers (decision functions), and the effects of each strategy on classifier error, in just such a situation.

### Website review—cancer post-genomics on the web

Cancer research is already benefiting from the application of post-genomics technologies. Several groups are already applying proteomics and transcriptomics to the discovery of targets for diagnosis and treatment. In this feature, Mark Albertella presents a guide to some of the best websites providing resources for cancer genomics research, giving us a guided tour of selected sites.

### Featured organism: reductive evolution in microbes, *Buchnera* sp., *Rickettsia prowazekii* and *Mycobacterium leprae*

Obligate intracellular bacteria commonly have much reduced genome sizes, compared to their nearest free-living relatives. One reason for this is reductive evolution: the loss of genes rendered non-essential due to the intracellular habitat. In this article we take a look at three such bacteria whose genomes have been fully sequenced. *Buchnera* is an endosymbiont of the pea aphid, *Acyrthosiphon pisum; R. prowazekii* is the causative agent of louse-borne typhus in humans; and *M. leprae* infection of humans leads to leprosy.

*The* Genomics *Website at Wiley*

www.wiley.co.uk/genomics

The Genomics website at Wiley is a DYNAMIC resource for the genomics community, offering FREE special feature articles and new information EACH MONTH.

Find out more about *Comparative and Functional Genomics*, and *Proteomics*, and how to view many articles FREE OF CHARGE!

Visit the Library for hot books in Genomics, Bioinformatics, Molecular Genetics and more.

Click on Journals for information on all our up-to-the minute journals, including: *Genesis, Bioessays, Gene Function and Disease, Human Mutation, Genes, Chromosomes and Cancer* and the *Journal of Gene Medicine.*

Let the Genomics website at Wiley be your guide to genomics-related web sites, manufacturers and suppliers, and a calendar of conferences.

WILEY

2171

Copyright © 2001 John Wiley & Sons, Ltd. 

